# Being Normal yet Different: A Qualitative Study on the Dualistic Experience of Living With Unilateral Cleft Lip and Palate

**DOI:** 10.1177/10556656221121037

**Published:** 2022-08-17

**Authors:** Anna Paganini, My Engström, Hans Mark, Martin Persson

**Affiliations:** 1 Department of Plastic Surgery, Institute of Clinical Sciences, 70712 Sahlgrenska Academy, University of Gothenburg, Gothenburg, Sweden; 2 Department of Reconstructive Plastic Surgery, Region Västra Götaland, 56749 Sahlgrenska University Hospital, Gothenburg, Sweden; 3 Institute of Health and Care Sciences, 70712 Sahlgrenska Academy, University of Gothenburg, Gothenburg, Sweden; 4 Department of Surgery, Region Västra Götaland, 56749 Sahlgrenska University Hospital, Gothenburg, Sweden; 5 Faculty of Health Sciences, Kristianstad University, Kristianstad, Sweden

**Keywords:** cleft lip and palate, qualitative study

## Abstract

**Objective:**

The aim of the present study was to describe the experiences of young adults living with cleft lip and palate (CLP) and to explore potential gender differences.

**Design:**

A descriptive qualitative study was designed involving semi-structured interviews. The interviews were analyzed using qualitative content analysis, as described by Graneheim and Lundman.

**Participants:**

A total of 9 women and 8 men, aged 22 to 26 years with UCLP.

**Results:**

The main theme identified was: the duality of living with a cleft—being normal yet different, and 2 subcategories: “My cleft and me” and “My cleft and the World.” The participants described themselves as normal yet different, both in relation to themselves and in relation to others. They also stated that gender norms regarding appearance affected their lives and how they saw the cleft.

**Conclusion:**

This study adds to the growing body of qualitative research on CLP. It highlights the dualistic experiences of feeling normal and different at the same time. The interviews indicated that this dualism was based on context and gender, showing the psychological complexity of an individual. The clinical implications of this study emphasizes the need of a person-centered care approach in the cleft care setting where the clinicians are aware of the potential dualistic experience that also may differ over time that individuals with cleft can experience. This can also help clinicians better understand and help patients reduce distress and strengthen positive coping mechanisms.

## Introduction

Cleft lip and/or palate (CL/P) is the most common congenital facial malformation, affecting approximately 2:1000 live births and is more common among males than females.^1,2^ A cleft lip and palate (CLP) affects function as well as facial appearance. A treatment program is usually provided by a multidisciplinary team that starts in infancy and extends into childhood, adolescence, and sometimes adulthood. It consists mainly of reconstructive surgeries to repair the cleft, together with speech training and orthodontics, with the goal of achieving the best possible functional and aesthetic outcomes as well as achieving psycho-social well-being.^3,4^

Many individuals with CL/P live “fruitful and fulfilling lives”,^5–7^ but others experience a substantial psychosocial impact on their lives. This impact may include appearance-related concerns,^4,8^ teasing and bullying,^5,6,9^ and problems with the remaining sequelae of the cleft, such as speech and hearing impairment.^10^ Several qualitative studies on adults living with cleft have described a feeling of not being normal^5,6,10,11^ and a longing to be like everyone else.^12^ In addition, some individuals with cleft may struggle well into adulthood, where the cleft keeps causing distress when entering the workplace, forming a romantic relationship, or starting a family.^13^

The impact of living with CL/P is lifelong and varies during the course of life^14,15^; it seems that for most individuals, the impact diminishes over time and a feeling of acceptance emerges together with a feeling that the cleft must not hinder life satisfaction.^5–7,14^ Dissatisfaction with appearance that impairs self-confidence seems to decrease during adulthood.^7^ Qualitative studies also describe that living with a cleft help can develop empathy, strength, and resilience.^11,16^ However, it is important that psychological and other support be available in adulthood,^13^ since the cleft might be a stressor that continually causes distress.^7,17^

Existing literature regarding the ongoing psychosocial impact of a cleft in adulthood is still limited, but it is steadily increasing. The influence of gender on the experience of living with a cleft has not yet been fully explored and so far, mainly discussed in relation to appearance.^16^ Differences between men and women have been identified in quantitative studies, such as with regard to psychiatric health,^2^ achievements in school,^18^ coping strategies, and appearance-related distress.^17^ However, no evident relationship between gender and psychosocial adjustment has been found^10^ and this area needs further exploration. There is a lack of knowledge with regard to how gender influences individuals with CLP and their well-being. This study adds to the growing body of international research and further the understanding of the experience of growing up and living with a unilateral cleft lip and palate (UCLP) from a gender perspective.

## Aim

The aim of the present study was to describe young adults’ experiences of growing up and living with UCLP and to explore potential gender differences.

## Methods

### Study Design

This study was designed as a qualitative inquiry; participants were interviewed using individual semi-structured interviews and data were analyzed using qualitative content analysis. The recommendations for qualitative research according to the COREQ criteria were followed throughout the research process.^19^ The COREQ checklist help researchers follow and report essential aspects of qualitative research both regarding research team and reflexivity, study design, data analysis and reporting, and it aims to increase transparency and quality of studies. The first author (AP), who conducted the interviews, is a specialist nurse in pediatrics and is experienced with cleft care. However, they were not involved in the care of the participants.

### Participants and Setting

In Sweden, multidisciplinary cleft care is freely available to all children born with cleft, and takes place in 6 tertiary CLP-centers, from birth to 19 years of age. The participants of the study were recruited purposefully from the roster of individuals born with UCLP in the region of West Sweden. The individuals had received care in the tertiary center of Sahlgrenska University Hospital and were born between 1993 and 1998, hence were 22 to 26 years of age at the time of the interviews. Exclusion criteria were known syndromes, language barriers as well as lack of information on current address. The age of 22 to 26 was chosen to ensure that the standardized cleft treatment protocol was finished for all participants.

Potential participants received a letter introducing the study and were contacted by phone a few days later. In total, 9 women and 8 men were interviewed. Three women and 12 men declined participation, mostly due to time constraints. Saturation was reached after 7 participants of each gender. An additional 2 women and 1 man were interviewed to confirm saturation, after which data collection was completed. The median interview time was 24.5 min (range 20-44) for men and 31 min (range 28-55) for women. Table 1 presents the demographic data of the participants.

**Table 1.** Participants Demographics.

	Male (n = 8)	Female (n = 9)
Age, median (range) years	24 (23-26)	24 (22-26)
Occupation		
Full-time work	5	4
Studying	2	5
Unemployed	1	0
Academic background		
University, finished or ongoing	3	6
High school diploma	4	2
Not finished high school	0	1
Living conditions		
Alone	4	7
With significant other	2	2
With parents	2	0

### Data Collection

The interviews were conducted in October and November 2020 via the teleconferencing system Zoom (Zoom Video Communications, Inc.) (*n* = 15) or telephone (*n* = 2) and the participants chose the format. The interviews were conducted in Swedish. All interviews were audio-recorded and transcribed verbatim by the first author, AP. The interviewer (AP) began with their introduction and that of the aim of the study. A topic guide was used, where the main questions were “Can you tell me about how it is to live with a cleft,” “Can you tell me about growing up with a cleft,” and “Do you think there is any difference between a woman and a man with a cleft.” Probing questions such as “can you explain” and “what do you mean” were used, in order to elicit details.

All patients were informed both at the start of the interview and in the written information material that they could receive follow-up support from the cleft team psychologist if needed. All participants received contact information of the team psychologist and 1 participant was referred by the researcher after the interview.

### Data Analysis

Data were analyzed using qualitative content analysis with an inductive approach, as described by Graneheim and Lundman.^20^ The analysis was performed in Swedish, and quotes were translated by a native English-speaker with a Master in Arts-degree.

First, the interview transcripts were carefully read several times to understand the content. Meaningful units that captured the essence of the participants’ experiences of living with CLP and gender norms were highlighted and condensed into brief phrases, which were labeled with codes using the qualitative analysis software NVivo 12 (QSR International). One interview was independently analyzed by AP and ME and then the analysis was compared to ensure validity. Thereafter, the initial analysis was performed by AP. Next, investigator triangulation was performed by ME and the codes were sorted into subcategories. Similar subcategories were grouped into main categories from which an overarching theme emerged. Triangulation was performed again by MP where some interviews were read in light of the coding to ensure the validity of it. Throughout the analytical process, findings were discussed among the authors to ensure agreement. Trustworthiness was ensured by measures of credibility, dependability, and transferability as described by Graneheim & Lundman.^20^

### Ethical Considerations

This study was approved by the Regional Ethics Board of West Sweden (approval no. 970-11). All participants received both verbal and written information before participating in the study and provided written informed consent. They were informed that participation was voluntary, that they had the right to withdraw from the study without having to specify the reason, and that confidentiality was guaranteed.

## Results

The interviews yielded deep and rich narratives from young adults with UCLP regarding their experiences. A latent overall theme that emerged from the data analysis was “The duality of living with cleft—being normal yet different” (Table 2). All narratives contained descriptions of experienced contrasts in life and the theme emerged explicitly.

**Table 2.** Findings of Content Analysis and Number of Respondents in Each Subcategory/Category.

Theme	The duality of living with cleft—being both normal and different					
Category	My cleft and me			My cleft and the world		
Sub-category	My appearance *(8 men, 9 women)*	My future *(3 men, 5 women)*	My thoughts *(8 men, 9 women)*	I am like everyone else *(8 men, 9 women)*	My relations *(8 men, 9 women)*	I am an outsider *(7 men, 9 women)*
Codes	Feeling unattractive *(2 men, 6 women)*	To plan for the future *(1 man, 3 women)*	It’s worse for others to have a cleft *(4 men, 5 women)*	I feel normal *(8 men, 9 women)*	My family and friends are important to me *(5 men, 8 women)*	The cleft is a burden *(7 men, 9 women)*
	The cleft affects my appearance *(7 men, 6 women)*	To meet someone *(2 men, 5 women)*	Difference between genders—or not *(6 men, 9 women)*	Time has healed me *(8 men, 9 women)*	Bullying *(4 men, 6 women)*	I feel different and as an outsider *(4 men, 9 women)*
	My teeth are a big deal *(6 men, 6 women)*		I cope with problems that arise *(3 men, 5 women)*			Societal norms *(7 men, 9 women)*
			Good things have come from the cleft *(8 men, 8 women)*			

The overarching theme consisted of 2 categories: “My cleft and me” and “My cleft and the world.” “My cleft and me” emerged from the manifest subcategories “My appearance,” “My future,” and “My thoughts,” which focused on the individual’s perception of themselves. “My cleft and the world” was built on the subcategories “I am like everyone else,” “My relations,” and “The cleft has made me an outsider,” which focused on the individual in relation to society.

### My Cleft and Me

*My appearance* was discussed in all the interviews, ranging from an awareness of the cleft’s impact on their appearance to feeling unattractive due to it. All participants acknowledged that being born with a cleft affected their appearance. Their experience ranged from positive—feeling more interesting—to negative—feeling unattractive. Often, several sentiments were present in the same narrative, capturing the complexity of their relationship with their appearance.

If I put a value on it, it would be positive. In that, I think that my face would have been more boring without… or that it is just an important part of my face. (F9)

A majority of the women, 6 out of 9, expressed feeling unattractive due to the cleft, which was not described by any of the men. Gender norms regarding how a woman should look were also attributed as affecting an individual’s feeling of being attractive.

It doesn’t feel more attractive to have a scar in the face of itself, it’s just less attractive then. I think. It is something one has pondered… you get to hear that /brother/ was cute with his scar, and then I came as an ugly child. My mother has said that there were comments like that. But yea… also, as a woman you should have a certain look, and you have norms and such, think also that how my self-image would have looked like. (F1)

Some individuals stated that their appearance was not very important to them, but they simultaneously acknowledged that appearance is important in general, which made them feel the need to “look nice.”

Ok…yeah because it feels like… I don’t know, maybe it’s a preconceived notion, but they… that there are kind of not that many with this condition who are fixated on their looks, and I’m not fixated on it either, but it’s not that I care a great deal about my looks. But I… you still want to look nice and… (F8)

There was a feeling of certainty among the participants that the cleft and the cleft-related appearance could have had a larger impact on their lives if it had been more visible.

Because when you have had a cleft lip *[and palate]* that is visible when you are a grown up you probably wouldn’t have had relationships as easily… and probably not gotten to live a normal life like I do. (M7)

Teeth were most often mentioned by the participants regarding appearance, regardless of gender. Concern was expressed regarding how teeth currently affect or had affected the individual’s appearance. Participants described how teeth are central to the face and that crooked or abnormal looking teeth made them feel self-conscious.

Yes, it’s sort of always been that my teeth are very very crooked. When I was younger, I was sort of… I thought of that almost all the time. You kind of pondered that my teeth are crooked and things like that. (M8)

*My future* was a subcategory that described thoughts and concerns regarding the future. Many displayed fear that the cleft would continue to influence their future adult life, just as it had in the past. Some individuals acknowledged that they had previously felt fear but had moved past it—either due to age and maturity or by meeting a partner.

A majority of the participating women, 5 out of 9, expressed worry about meeting a partner and having children. The fear regarding children encompassed both the fear of not having children and also a fear of having children with a cleft, who are forced to experience what they had endured.

But I am also sort of scared for… I am a little sceptic to have children of my own that has cleft lip, cause I don’t want them to go through that bullying and such, but I feel if I have that its mostly to find …. I am mostly worried about finding a partner that would want me, but not my children with cleft palate. It is also a thing that one ponders. (F1)

The men did not discuss future children; instead, their narratives contained more discussion about meeting a significant other. The cleft was not as important in their narratives regarding serious relationships and they were overall more positive regarding meeting someone than the women. A greater sense of contentment was found among the men.

Well relationships, for example, I’ve never had any problems getting girlfriends actually. Even before I had a beard. My CLP obviously shows less when I have a beard, but even when I didn’t have a beard, I have never had any problem with relationships and things like that. (M7)

*My thoughts* was the third subcategory, describing the participants’ perception of their cleft and its influence on their life as well as a comparison with others with cleft.

Half of the men, 4 out of 8, claimed that they had been lucky compared to others with a cleft, who had been less fortunate and were worse-off both in terms of having peers with cleft in Sweden or being born in a country with access to affordable health care.

Then, I’m very fortunate, I’m in a good seat. I have a permanent job, I have an amazing girlfriend, I have a great life, have my family, I kind of can’t complain. (M7)

When reflecting upon whether it was harder to grow up as a girl or as a boy with a cleft, many women described that it probably would be easier to be a man since it is possible to hide the scar with a beard. Conversely, several men mentioned that women have an easier time hiding the cleft with makeup. Most participants, however, thought that besides the gender norms regarding appearance, there was no difference between the genders regarding living with or growing up with a cleft.

Several different ways of coping with the cleft and feeling different emerged. In cases of speech impediments and physical problems involving food and drink that enter the nose, most individuals described an improvement over the years. The improvement had come through practice, either with the help of a speech and language therapist or because of their own work. Other active ways of coping included being open and transparent about one’s difficulties, for instance, in situations where others have problems understanding the individual with a cleft.

Well, I’m used to it, when I meet new people, I usually say straight away that if you don’t hear or if you don’t understand, just ask or what not. (M3)

Some participants stated that they tried to avoid situations where the cleft might be noticed or questioned.

Ehm…but I also think that I avoided such situations, where you could be criticized for your looks and such. (F8)

In some cases, more drastic coping strategies were used to avoid bullying and socially difficult situations. One man described a voluntary isolation during the teenage years to avoid tough situations.

I sort of ended up going to the computer as a haven and kept mostly to myself indoors. (M7)

All but one of the participants mentioned that there were benefits of living with a cleft. Some individuals talked about practical advantages, such as the cleft could function as a subject for class presentations or being able to attend special events with celebrities through the hospital and charities. Other positive experiences included personality growth due to the impact of living with a cleft or increased self-awareness with the help of a psychologist at the center. Several individuals mentioned that the cleft and the experiences of growing up with it were important reasons for choosing a career in public service.

The ability to stand up for yourself, I think … because it really has led to the person I am today, the job I have. (M7)

### My Cleft and the World

The participants not only reflected on themselves but also about the world. The category *My cleft and the world* focused on the participants’ relationships with others and how they viewed themselves. This category had its foundation in the subcategories “*I am like everyone else,” “My relations with others,” and “Being an outsider.”*

*I am like everyone else* emphasized the feeling of normality among participants. All 17 participants discussed feeling like everyone else, but from different points of view; first, from a family perspective, when several people in their family had a cleft, it made it quite normal.

It is like this, the majority of the people in my family have cleft lip and palate, and from the family perspective its quite normal. (F1)

Second, people also described feeling like everyone else, meaning that living with a cleft had never been extraordinary. The cleft was a part of the individuals’ life and in relation to their surroundings, that is, their normality.

I have sort of not really thought about it, kind of like this, that is something different, cause it’s really never been a big thing. (M4)

Another finding was that this feeling of normalcy had developed over time and the transition from youth to adulthood helped participants come to terms with the cleft.

But I don’t think about my cleft as I did when I was younger. Now, it feels more like a part of me. (F5)

*The cleft has made me an outsider* was found in all narratives but one and had more or less negative connotations. The participants described a feeling of being different than others, which emerged as a distinctly dualistic experience given that participants simultaneously described themselves as “normal.”

And I think that, I may not have gotten as many comments about it (*the cleft*), but still I have that feeling *(of being an outsider).* So I think that people that receive more comments about that, really have a stronger feeling*.* (F7)

This feeling of being an outsider was sometimes considered a strength, as a feature that not everyone has. To be different, is not always a flaw.

But… you have learned that, you are not like everybody else. In a way, you kind of like that. So you don’t end up this typical… where everything has to be perfect and such. (M3)

One gender difference found was that the women described the cleft as a burden to bear, particularly in the past, but also at present. Such a negative perspective was not found in the narratives of the men.

I feel that it has been very hard. It’s something that I feel… It made me feel really bad about it to be really honest. It was not very long ago, that I got all broken up about it, and kind of didn’t want to be here anymore. I started to wonder why my parents didn’t do an abortion and such. But that they let me…. I feel that I suffer every day. So that’s tough. (F3)

Participants also discussed how societal norms affected their view of appearance. Several women said that gender norms added to the feeling of being an outsider and different, since they did not look the way a woman should. It was described that a man can have a scar without facing social repercussions, something that is difficult for women. One participant explained with an example of a celebrity with a cleft.

You know the guy actor Joaquin Phoenix ehh.. there is… it feels like he has become so… he has such a characteristic face cause of the cleft lip scar. But I have a hard time imagining that an actress in her forties… “She has a very characteristic scarring there” (F9)

There was a consensus among the participants that norms regarding appearance are stricter for women than for men and that they put pressure on the individual to be a certain way.

But as everyone knows, girls have higher pressure on their appearance, it feels like. Over all, compared to guys. (M6)

*My relations* was a subcategory that all participants reflected on from different perspectives. The importance of family was described along with the sense of safety that it offered during childhood and youth.

But it’s still nice that the family has been a safe haven so that one has been able …. And my grandmother was very much on standby and came to help out. (F1)

Having friendships during youth and childhood was of great importance to the participants that had a protective function for the individual to avoid bullying and also to feel included in the social setting. About half of the participants, both men and women, described being subject to bullying during their youth and childhood, together with not having close relationships with friends, creating a feeling of loneliness.

Ehh…it was pretty hard, because I was bullied just because I was different and such. Eh… so you were kind of… no, it WAS hard to get new friends and such. Eh… so you were kind of lonely. (M3)

In all cases, bullying was described as part of the past and all affected participants explained that in the present they no longer experienced it. However, the residual effects of being bullied were present in some of the narratives and included fear of meeting new people and a constant sense of vigilance to avoid situations that could potentially lead to being bullied again. Several participants expressed a fear that meeting new people might trigger difficult situations and that the cleft would be the first thing that new friends and acquaintances would notice.

Ehh… its the same when you meet new people… like now, for example, when I went back to school and was meeting my classmates for the first time. In my head, the cleft palate is always the prominent thing. That this is what they will notice first about me. /silence/ (F4)

This vigilance is present when interacting with others, even though most of the participants are well aware that the people around them generally do not notice the cleft.

But… if I don’t mention it to people, they don’t seem to notice. (M2)

## Discussion

This study aimed to describe young adults’ experiences of living with a UCLP. The participants were aged 22 to 26, which placed many of them in the transition period called emerging adulthood.^21^ In this period, young adults develop their identity before committing themselves to long-term choices in adulthood.^21^ The participants were in the process of becoming adults, which included some having a secure job and a significant other, while others were still living at home and recently started university or vocational training.

**Figure 1.**
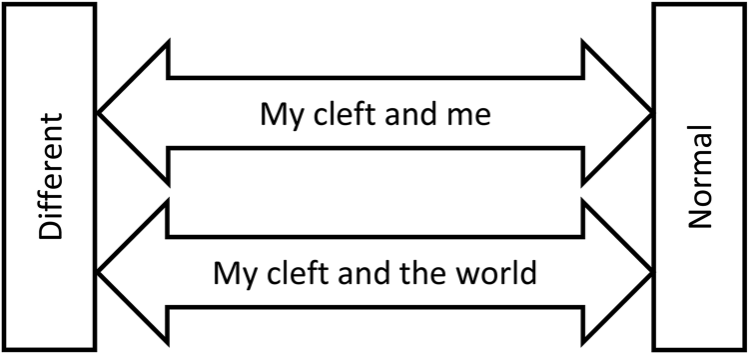
The duality of living with a cleft lip and palate: exploratory model.

The main conclusion from our data is summarized in an overarching theme: *The duality of living with cleft—being normal yet different*. The respondents stated that they felt very normal, yet very different; these feelings were relative and differed over the course of life and in different situations, mirroring the dynamics of life. This study yielded an exploratory model, as shown in Figure 1. It was clear that for a majority of the participants most aspects could be both positive and negative, or somewhere in between, showing the complexity and dynamics of life. Participants identified the feeling of being different along with an equally strong sentiment of being like everyone else. Some individuals felt intensely different than others, while others more strongly claimed normalcy, demonstrating a diversity of narratives and individual variation. The diversity among individuals living with a cleft has been substantial, where positive, negative, and neutral responses to the same concept have been described.^11,16^ This was confirmed by the present study, with the added knowledge that most participants encompass both feeling normal and different in varying degrees during different life circumstances and situations. There were no obvious gender differences present, but women had a more significant negative connotation in their narratives, describing their cleft as a burden.

The feeling of being normal was described in the present study and was often put in a context. Several participants talked about feeling normal in their families or with their friends in contrast with feeling different compared to the general public, showing the contextual nature of feeling normal. Several previous studies have shown that a pursuit of and longing for normalcy is present among adults with CLP^5,6,10–12^ and that the perception of difference may be the key to understanding social experiences among individuals with cleft.^16^ No gender differences were found regarding this. This study adds that normalcy and difference may be present at the same time in an individual with CLP, but differ in intensity based on the individuals’ previous experiences, age, and the social setting they presently are in.

In some narratives, it was described that the physical sequelae from the cleft enhanced the feeling of being different, for instance, when the participant’s speech was impacted by the cleft. However, having impacted speech also created the need for coping strategies, such as actively letting the other person in the conversation know they can seek clarifications. Previous qualitative research, focusing on communication and speech, described that one way for individuals with CLP and speech problems to cope is to take charge of their communication.^5,6^

Other difficult situations such as teasing and bullying during childhood and adolescence were also described. Individuals of both genders were bullied but it was more commonly described by the women. Being bullied enhances the feeling of being different and an outsider, as well as having long-term psychosocial impact.^22^ Several types of coping strategies were used to deal with bullying, such as finding safety among family and friends, but also by self-isolating and fleeing situations. Different coping strategies by individuals born with a cleft in relation to gender have previously been explored and identified in a quantitative study,^17^ which was also indicated in the present study.

Appearance was also discussed and it was clear that it was a major contributing factor in feeling different. Awareness of the cleft’s impact on appearance was described as well as feeling unattractive due to the cleft. Appearance was seen as the gateway to normality and participants expressed that they thought that the visibility of the cleft and the cleft size impacted their normalcy. This clearly shows that the influence of subjectivity on appearance is paramount however, literature has not found any correlation between cleft size and the psychosocial impact of life.^23–25^ It has previously been described that individuals place different levels of value on their appearance and those who characterize their appearance as highly important also seem to be more concerned with cleft-specific appearance differences.^16^

The women in the study expressed more concerns about feeling unattractive due to their cleft than men. A contributing factor might be the influence of societal norms regarding appearance may be stricter for women, something all participants, regardless of gender, expressed awareness about. It was also described that women face more pressure regarding their appearance than men. The literature supports this statement,^26,27^ however, appearance-related distress among men is steadily increasing.^28^ Future research should investigate appearance-related distress among men with CL/P and if the mechanisms behind it differ from women.

Male and female experiences of growing up with UCLP were similar and the variations were more pronounced within each gender than between them. However, some differences between genders could be identified. The women in the sample had more thoughts and worries about their future, especially in terms of starting relationships and having children than men. Many women had thought about how the cleft would influence them both in life and in starting relationships. Overall, the men looked at the future more positively and the cleft was not as important in their narratives. Men were also more likely than women to feel lucky or better off than others with a cleft. It has been shown that men have a higher level of dispositional optimism than women^17^ and the results of this study could be interpreted in this light. However, further studies are needed to explore this.

The described gender differences found among the participants, but also the similarities found between genders, creates a possibility to better provide gender-sensitive psychosocial support from the cleft team to patients.^29^ Future studies are needed to better create gender-sensitive cleft care.

### Study Limitations

Some limitations of this study must be acknowledged. The intention in the study design was to approach all individuals with UCLP, aged 22 to 26 years, being treated at the same center. However, since participation was voluntary, the sample cannot be assumed to be representative for the whole group of young adults with UCLP. In the present study, all but one of the participants were working or studying at a secondary level; this may be an indication of a well-adjusted population. Furthermore, there is a need to include diverse cultural groups and groups with lower health literacy to address the health inequalities. This has previously been noted^16,30^ and the findings of present study must be cautiously understood in light of the relatively homogenous participants.

The interviews were held via the videoconferencing system Zoom, a methodology that has not yet been validated in larger studies. It has several advantages compared to other online videoconference systems, such as it does not require an account or a downloaded program and can be password-protected for confidentiality.^31^ In this study, the recording capacity of Zoom was not utilized, to ensure a high level of confidentiality. All but 2 participants chose to participate through Zoom, and the individuals who chose telephone interviews stated technical problems or network issues. Telephone was used as a back-up, in accordance with the recommendations of Gray et al.^31^ The Zoom interviews allowed the participants to be comfortable in an environment of their own choosing, providing a sense of security and still feeling personally connected with the interviewer.^31,32^ Finally, when attempting to execute qualitative research over long distances and during a pandemic, Zoom is a functional resource.

## Conclusion

This study adds to the growing body of qualitative research on individuals with CLP. This study contributes to the dualistic experiences of feeling normal and different at the same time. The interviews indicated that this dualism was contextual and gender-dependent, showing the psychological complexity of an individual. The clinical implications of these findings emphasize the need for a person-centered care approach in clinical settings, ensuring an awareness of the dualism that individuals with a cleft can experience. This can help clinicians to better understand and help their patients to reduce psychosocial distress and strengthen positive coping strategies.
